# “Womanhood,” a shared experience of participating in a lifestyle intervention with a focus on integration and physical activity to promote health among pregnant women: perspectives from pregnant women, midwives, and cultural interpreter doulas

**DOI:** 10.1080/17482631.2022.2043527

**Published:** 2022-02-25

**Authors:** Nina Malmström, Marie Lydell, Ing-Marie Carlsson

**Affiliations:** Department of Health and Welfare, Halmstad University, Halmstad, Sweden

**Keywords:** Doulas, foreign-born, integration, lifestyle intervention, physical activity, pregnancy, reproductive health, qualitative studies

## Abstract

**Purpose:**

Migrating women, have an overall increased risk of adverse outcomes and poorer health during pregnancy and childbirth. In addition, they do not participate in planned antenatal care to the same extent as natives. These disparities among migrants and native pregnant women point to the need for interventions to improve equal health and care during pregnancy and childbirth. This study aimed to explore the experiences of participating in a lifestyle intervention, named “Dancing for birth,” focusing on integration and physical activity, from the perspectives of the participating pregnant women, midwives, and cultural interpreter doulas.

**Method:**

Qualitative interviews were conducted from March 2019 to December 2020, with ten women who participated in a lifestyle intervention in Sweden: four pregnant women, three midwives, and three cultural interpreter doulas. Thematic analysis was used to analyse the data.

**Results:**

The lifestyle intervention ”Dancing for birth” provided positive shared Health-promoting experiences among the participants with social inclusivness and a commitment to supporting each other. This seemed to encourage the sense of strength as a woman- a strengthboth for the individual woman and as a kind of women´s power.

**Conclusions:**

Interventions targeting physical activity, social inclusiveness, and health literacy are of utmost importance in promoting positive pregnancy experiences and equal healthcare during pregnancy. Further research is needed on how to implement antenatal education that includes all women in society.

## Introduction

It is of utmost importance that maternity care meets the needs of all women in society and care should be based on an individual assessment of the needs of every woman. This poses challenges since immigration to Sweden has increased, and among this population of foreign-born, a proportion are women of reproductive age (Swedish Central Bureau of Statistics, 2020).

Today, more than one in five pregnant women are born in a country outside Sweden. This increase is of utmost importance for public health because previous research has shown that migrating women may have increased risks of adverse pregnancy outcomes as compared with native-born women (Henriksson et al., 2020; Seghieri et al., 2020).

An independent risk factor for maternal and neonatal morbidity and mortality is pre-pregnancy obesity (Guelinckx et al., 2008). A large Swedish population-based registry study showed that the prevalence of obesity in the first trimester is higher among migrant women than native-born women (Henriksson et al., 2020). These results are in line with previous European studies (Reiss et al., 2015; Torkildsen et al., 2019). Maternal obesity is associated with a higher risk of gestational diabetes, hypertension, preeclampsia, and adverse birth outcomes with increased risk of caesarean delivery and wound complications (Catalano & Shankar, 2017; Marchi et al., 2015; Melchor et al., 2019). Moreover, maternal obesity is also associated with adverse outcomes for the child with premature birth, delayed delivery, large children, stillbirth, and malformations in the child (Catalano & Shankar, 2017: Melchor et al., 2019). In addition to obesity, depressive symptoms, and the incidence of suicide during or after pregnancy is higher among migrant women as compared to natives (Esscher et al., 2016; Hultstrand et al., 2020).

Health inequalities among migrant pregnant women stem from several factors and are complex, as health and integration interact (World Health Organization, 2018). Studies on migrant populations have shown that factors included are the process of migration, exposure to risks and access to the determinants of health in the country of origin (World Health Organization, 2018). Moreover, previous studies support that migration and adverse birth outcomes varies by migrant subgroup (Akselsson et al., 2020; Gagnon et al., 2011; Juarez et al., 2017; Urquia et al., 2010), with women from low-income countries being particularly vulnerable, having a 6.6 times higher maternal mortality rate compared to Swedish-born women (Esscher et al., 2013). Moreover, the destination country and the social circumstances that immigrants encounter in the new country affects immigrant’s health and adoption of unhealthy behaviours (Lindström & Sundquist, 2005; Lopez-Borbon, 2021). While the reasons are multifactorial, it is worth recognizing that small life-style changes in pregnancy can have a substantial impact on maternal and child health (Stephenson et al., 2018).

Physical activity during a normal pregnancy has been shown to have several positive health effects for the pregnant woman, both mentally and physically (Gaston & Prapavessis, 2013; Rodriguez-Blanque et al., 2019). Regular physical activity has been established to be an important factor in promoting balanced weight gain, but also in preventing and, to some extent, treating overweight and obesity (Sagedal et al., 2017). Despite this, women in general, but especially foreign-born women, exercise less during pregnancy than before and many are even physically inactive (Hultstrand et al., 2020; De Vivo & Mills, 2019).

Antenatal care (ANC) has significant potential to change health behaviours and improve health. In Sweden ANC is provided at community-based clinics and is free of charge. Midwives are the main providers of antenatal care, with a referral system to obstetricians when needed (Stephansson et al., 2018). The national guidelines recommend a minimum of eight visits during pregnancy; almost all women attend but not to the same extent. Research findings have demonstrated that foreign-born women in Sweden have lower antenatal care attendance compared to natives (Berggren et al., 2006; Ny et al., 2007).

In Sweden, parent education including preparation for childbirth and parenting is provided throughout pregnancy, also free of charge. However, education often focuses on first-time parents and is held mainly in Swedish, which leaves foreign-born women to rely only on individual information given by the midwife. Consequently, foreign-born women express that maternity care is not adjusted to the women’s needs according to language and communication difficulties and unmet perinatal information needs (Balaam et al., 2013; Fair et al., 2020; Lyberg et al., 2012; Small et al., 2014). In addition to language and communication difficulties, an inadequate use of interpreters within the healthcare system has also been described (Balaam et al., 2013; Small et al., 2014).

However, in some parts of Sweden, a doula can be offered at ANC (Akhavan & Edge, 2012). A doula is a trained professional who provides physical, emotional, and educational support to women before, during, and immediately following childbirth.Further, cultural interpreter doulas can offer multilingual and cultural competence. Previous research describes the assistance of a doula as important support and states that it is associated with healthier birth outcomes, with fewer medical interventions for both the mother and the child (Akhavan & Edge, 2012; Gruber et al., 2013).

In 2018, a pilot project, with the lifestyle intervention “Dancing for birth,” was initiated as a model for safe and equal care, with a focus on foreign-born women. The project was initiated, designed, and performed by midwives at ANC and held in a region in the southwest of Sweden with the aim of supporting and strengthening women during pregnancy and preparing for childbirth both physically and theoretically. The project was implemented in cooperation with the non-profit and non-governmental association Aligi, which organizes doula and cultural interpreter courses in the southwest of Sweden, and the Administration for Culture and Health. It was financed by the Swedish government based upon an agreement with Sweden’s municipalities and county councils (SKL) to ensure that all expectant and new parents feel safe and secure throughout the process, before, during, and after pregnancy.

The pilot project lasted for twelve weeks from September to December 2018. The main target group for the project was foreign-born women, but Swedish-born women were also invited to participate to open for integration. The women could join at any time during pregnancy. The recruitment was conducted at antenatal clinics via oral information from the midwifes and written information in the form of brochures and posters translated into several languages. Written information was also available at libraries, the premises of Swedish for Immigrants (SFI), and other environments where pregnant women were expected to be. The intervention team consisted of three midwives, one of whom had been assigned the role of group leader, a dance instructor, and five cultural interpreter doulas. The doulas were trained health workers and some of them were also educated midwives in their countries of birth. In addition, they helped with the language during the group sessions of the lifestyle intervention and translated when needed.

The lifestyle intervention consisted of a group session once a week; everything was free of charge. The sessions had three components: physical activity, antenatal education in a group, and the goal of increased integration into society. During each session, a dance class led by a trained dance instructor was held, with the focus being on increasing physical activity during pregnancy. It was a soft liberating dance, specially adapted for pregnant women, with a focus on creating a good feeling in the body and a joy of dancing. Everyone could participate based on her own condition. The dance was followed by a group conversation about childbirth and parent preparation and then a break, when coffee, tea, and fruit were served. On one occasion, the National Dental Service was invited to give a lecture, and on another occasion, a dietitian was present. The entire group of women who were present—the pregnant women, the doulas, the group leader, the two other midwives, and the dance instructor—participated in all the activities during the sessions.

According to Schytt et al. (2020) pregnancy interventions to improve maternity care for foreign born women should include activities that break down language barriers and build cultural understanding, increase familiarity with and understanding of Swedish maternity care, and increase women’s sense of safety and confidence in giving birth (Schytt et al., 2020). In addition to Schytt et al. (2020) recommendations, physical activity was added as an intervention in this project. To our knowledge there is a gap in research that have used dance as a physical activity during pregnancy. Given the vulnerability that pregnancy and childbirth can entail for foreign-born women’s health and health behaviour, innovative targeted interventions are needed, to be able improve care and health outcomes. Therefore, this study aims to explore the experiences of participating in the lifestyle intervention “Dancing for birth,” focusing on integration and physical activity, from the perspectives of the participating pregnant women, midwives, and cultural interpreter doulas.

## Materials and methods

### Study design

This study had an explorative design in which the empirical material consisted of qualitative semi-structured interviews. The method used was a thematic analysis according to Braun and Clarke (2019) with an inductive approach.

### Participants

To select participants for this study, purposive sampling was applied. The focus was on finding participants who were assumed to contribute with the shared experience according to thematic analysis and in relation to the purpose of the study (Braun & Clark, 2019). All seven pregnant women as well as the five doulas who took part in the lifestyle intervention were asked to participate in this study. The recruitment was conducted by the group leader, who informed the participants about the purpose of the study. A total of four women—two foreign-born and two Swedish-born—who participated during their pregnancies chose to be interviewed, as did three of the doulas. In addition, the midwife who was given the responsibility for the project and the two other midwives who actively took part in the project participated in this study. The doulas were foreign-born, while the midwives were natives. All ten participants were women between the ages of 26 and 49 years.

### Data collection

Data were collected between March 2019 and December 2020. Eight interviews were conducted: seven individual interviews and one group interview. The four women who took part in the lifestyle intervention during their pregnancies participated in individual telephone-based interviews one to six months after giving birth. The group leader participated in an individual physical interview a couple of months after the end of the intervention. The three doulas were also interviewed physically and at about the same time, but in groups. This was motivated by the perception that it would create greater security for the doulas to be interviewed together in groups and that the conversation would develop more like this than it would in individual interviews. The two other midwives were interviewed individually via the Zoom application due to the prevailing circumstances arising from the COVID-19 pandemic. The interviews were conducted by three different interviewers using semi-structured interview guides with broad questions as support and lasted between 15 and 58 minutes. Examples of questions asked of the participants are: “What opportunities did you see with the project?”, “What were the main benefits of the project?”, “What do you think the project meant for the women involved?”, and “How do you see that this kind of project can promote women’s health and lifestyle during pregnancy?” All interviews were recorded using a digital voice recorder and then transcribed word by word, excluding the information that could somehow identify the participants.

### Data analysis

Braun and Clarke (2019) have divided the process of thematic analysis into six steps: familiarization, coding, generating themes, reviewing themes, defining and naming themes, and, lastly, writing up. The six steps were followed during the data analysis of this study.

The analysis began with the first step, familiarization with the collected material. The data was read through several times. Some initial reflections were also observed and noted during the reading. The second step was to systematically extract codes from the data material. All interviews were coded manually and processed systematically to ensure that no data had been missed. When the coding was complete, all codes were reviewed, and any duplicates were removed. This was performed by the first author, but with continuous reconciliations with the other authors to increase the quality of the coding and to control consistency.

In the third step, the codes were analysed and organized into different groups according to patterns of content. Then a refined sorting of the codes was done to distinguish sub-themes and create tentative themes. In the fourth step, the given themes and sub-themes were reviewed, refined, and revised as needed. They were all verified in relation to the coding, the transcribed text, and the purpose of the study to ensure that the findings and the data were consistent. This analysis step was performed by all the authors together, as was the fifth step of defining and naming the themes. In the sixth step, each theme’s content was written as an aggregated synthesis, including quotes from the participants (Table I).

**Table I.** Examples of the analysis

Extracted statements	Code	Sub-theme	Main theme
“And then just to sit down and be a part of a context, to feel welcome and included … it’s worth every penny.”	To be part of a context	Belonging in the new community	Belongingness
“And just to do something together in a group, I think it is a lot of fun, and you can also make some new contacts. And even if you do not have much in common or know each other from before, you are all pregnant, and then there is always a lot to talk about.”	Pregnancy becomes the commondenominator	Relatedness to other women	
“It was all so positive. Everyone was so expectant, and everyone was so happy. It takes so little sometimes to make you feel welcome, and to make something fun.”	Happiness	Cultivating positive emotions	Joyfulness
“It’s nice with these small groups, you can talk to others in the same position and get some exchange, you all have something in common. Some had given birth before and some were worrying about various things. It was nice to be able to support each other. It gave me a lot.”	Nice to support each other	Women supporting women	Committedness

Finally, in total, seven sub-themes and three main themes were generated. Taken together, an overall theme was generated which permeated the participants shared meaning of taking part in the lifestyle intervention. In this study, the analysis process was not linear, which the thematic analysis does not require (Braun & Clarke, 2019). Themes and sub-themes were changed several times before the final version was determined.

To clarify the authors’ pre-understanding, the first author was a master’s student in health and lifestyle, and the co-authors had professional backgrounds as a midwife and a physiotherapist.

### Ethical considerations

The Swedish Ethical Review Authority approved the study, dnr. 2019–00251(2018-89). The study met the ethical guidelines found in the Declaration of Helsinki for Research on Humans (World Medical A, 2013). The authors din not take any part of the project design or in conducting the intervention.

## Results

### Womanhood

The thematic analysis resulted in an overall theme: “Womanhood,” which permeated the shared experience of participating in the lifestyle intervention among the women. Participation was perceived as providing opportunities for the participants to come together with other women from different groups of society and having different professional backgrounds and ethnicities, with pregnancy being the common denominator. This created a shared experience of being united in harmony. Being united increased a sense of belonging to other women and a commitment to supporting and strengthening each other in a joyful way. The health-promoting informative group conversations, in combination with the liberating dance, seemed to encourage the sense of strength as a woman—a strength both for the individual woman and as a kind of collective women’s power (Table II).

**Table II.** Overview of the overall theme, main themes, and sub-themes describing the experiences of the lifestyle intervention “Dance for birth” with a focus on integration and physical activity

*Overall theme* **Womanhood**		
*Main theme* **Belongingness**	*Main theme* **Joyfulness**	*Main theme* **Committedness**
*Sub-theme* Belonging in the new community	*Sub-theme* Cultivating positive emotions	*Sub-theme* Women supporting women
*Sub-theme* Relatedness to other women	*Sub-theme* Finding a joy being physical active with others	*Sub-theme* Knowledge sharing
		*Sub-theme* Cultural exchange

The shared experience of womanhood was based on three main themes: “Belongingness,” “Joyfulness,” and “Committedness” These were, in turn, sustained by seven sub-themes related to the participants’ experiences: “Belonging in the new community,” “Relatedness to other women,” “Cultivating positive emotions,” “Finding a joy being physical active with others,” “Women supporting women,” “Knowledge sharing,” and “Cultural exchange”. The themes are described below and illustrated by quotes that are characteristic of each theme.

### Belongingness

#### Belonging in the new community

The participants expressed how they enjoyed being part of a group during the intervention, and the word “community” was often brought up during the interviews. Belonging to a community meant that they had a feeling that they mattered to one another and that they were significant and needed. Several of the pregnant women explained how the experience of belonging in a new community gave them a feeling of being included and involved, which was highly valued. The atmosphere in the group sessions was described as an accepting, embracing, warm, and welcoming environment, created by them all together. The quotation below describes how a pregnant woman expressed that she found it easy to join the group the first time she participated.

They welcomed me very well, so I felt very happy and laughed and talked.

(Pregnant woman)

The midwives, in turn, expressed how they perceived the experience of belonging to a community as something emotionally charged with positive feelings and very valued for both the pregnant women and them. The midwives also emphasized how the intervention, in general, seemed to create a sense of coherence, especially for those women born abroad, through the meeting from other groups in Swedish society.

The major benefits of the project were probably … yes, the community. To integrate the women, invite them, make them feel welcome.”

(Midwife)

Being part of the project was described as creating a sense of belonging not only among the pregnant women but also among the doulas. The experience of being an important member and a part of a context that was doing something vital to contribute to other women and to Swedish society, as well, strengthened their sense of belonging and valued involvement. This was also confirmed by the midwives who stood next to them and watched the doulas’ self-confidence grow.

I think it (the project) is much needed, it is a great project and what we have done is important and good for everyone. For women, for the staff, for us, and that we contribute to society. I want to do that. I don’t want to sit at home and wait for help from Social Services, no. I want to become a member of Sweden, which is great.

(Doula)

#### Relatedness to other women

The intervention seemed to create a social space where the participants had the opportunity to gather and interact with other women. The pregnancy became the common denominator, and this seemed to bring them all together and create a sense of relatedness. Although they were different in many ways, the pregnant women appreciated meeting other women with whom they could identify, and who were undergoing the same process as them.

Pregnancy is very good in that way because we have something in common, and women that you usually had not talked to and/or hung out with—now you can do that because you are pregnant, and you share something with each other.

(Midwife)

In addition, coming together with other women also meant an opportunity to have someone to talk to which the pregnant women described as important and a need that the project could fulfill. Some expressed a need to talk to others in similar situations as well as to share their own experiences. Most pregnant women mentioned that these personal meetings itself was a great benefit for everyone involved, allowing for connections to people whom the participants would not have met otherwise. This was confirmed by the doulas, who also explained that everyone involved got something out of the cultural meeting, regardless of origin, and that this brought the women closer to each other. The Swedish-born women specifically referred to the meeting with the foreign-born women, and the foreign-born women, in turn, said that they thought it was fun to meet Swedish women.

It’s a very social thing, and you meet people you would not have met otherwise.

(Pregnant woman)

The opportunity to meet and connect with Swedish women was something that the midwives experienced as being important for the doulas as well.

### Joyfulness

#### Cultivating positive emotions

All participants, the pregnant women as well as the midwives and the doulas, described participation in the project as an enjoyable time that gave them pleasure, with a lot of laughter. They appreciated the opportunity to do something fun with other women and described how they enjoyed talking with each other, laughing together, and dancing together.

We laugh, we talk, we dance.

(Doula)

The joy of dancing was also something that all participants shared, and this was a moment that they had exclusively on their own, just for themselves, which they appreciated. The midwives believed that the pregnant women enjoyed receiving a little extra care and that this extra attention made them feel seen. The pregnant women expressed how participation in the intervention made them happy and helped them fight loneliness. The women said that having someone to talk to made everyday life a little less lonely.

I felt more happy and not so lonely when I took part in the project.

(Pregnant woman)

#### Finding a joy being physical active with others

The dance was described as something positive and joyful. In particular, the pregnant women described how it felt good to be physically active and to use their bodies. The dance was described as a pleasant way to move, though one of the pregnant women reported some pelvic pain the day after. One pregnant woman described the variation in the movement pattern compared to her normal one as being positive. The dance was also experienced as creating a relaxing effect by both pregnant and nonpregnant women and was described as liberating. Everyone expressed how it was a fun way to move; the dance made them happy and the music put them in a good mood. One of the midwives experienced how the music, as well as the movements, strengthened both body and soul, and how it had a positive impact on self-confidence.

Then we danced and it was a very pleasant way to move. You do not move much at the end of the pregnancy, you mostly just walk. So it was nice to do something else and she was very good, the instructor, and she made sure that everyone kept up and that it was important making it fun.

(Pregnant woman)

Everyone who participated in the project considered physical activity to be one of the most important parts of the project for pregnant women, especially for the foreign-born women. The doulas discussed how the economy, cultural factors, and a lack of knowledge can be obstacles to achieving a healthy lifestyle and how this kind of intervention can help to overcome those barriers. A pregnant woman said that she wanted to start exercising earlier in her pregnancy, but that she did not know how because, as a non-Swedish speaker, she had problems finding information.

In my culture, you get pregnant and then you have to sit and rest and not exert yourself. But when I went there (participated in the intervention), I was very happy because you danced and laughed and talked, and when I danced my body reacted well. So, for me, it was a lot of fun. Before I went there, it was very difficult for me (to exercise)—how should I do [it] the first time? But when I went there, I had a great time.

(Pregnant woman)

Furthermore, the foreign-born pregnant women experienced how the exercise in the intervention had positive effects on the birth. There was a consensus among the pregnant women that they would have liked to have participated in the intervention more frequently and to have started earlier in their pregnancies; the dance was the main reason for this. The midwives described the dance as being a useful tool for promoting physical activity as well as health and lifestyle among pregnant women. They also highlighted how these kinds of activities could increase motivation for physical activity.

### Committedness

#### Women supporting women

The environment where the intervention took place was experienced as a space where all participants were committed to supporting and strengthening each other. The fact that only women were present contributed to a feeling, among the participants, of being in a safe atmosphere, i.e., safe in terms of being in an environment where everyone cared for each other. The informal conversations during the group sessions were described as “*a warm, woman-to-woman talk*,” with a collaborative venture that focused on communicating positive birth experiences and trust in birth.

Some of the pregnant women expressed that it felt good to support each other in the group in different ways and that it produced a very positive return. One of the pregnant women, who was expecting her second child, expressed how she could calm the other pregnant women, and maybe even reduce anxiety among them, by sharing positive experiences from her previous birth.

The women communicated the value of commitment from the doulas and the midwives, who all participated actively during the conversations and shared their own positive experiences with pregnancy and birth. This personal commitment from them was seen as contributing to an environment of open communication, which also enabled and encouraged the pregnant women to take part.

… And that they (the midwives and the doulas) had so many different experiences and they were so open to share. They could just as easily have been quiet and just let us talk. They gave a lot of their own and what they went through during their births. It encouraged us to dare to ask questions or something. It created an open environment, permissive.

(Pregnant woman)

The doulas, as well as the midwives, stated several factors that influenced the meaningfulness of their work and engagement at the intervention, such as doing something important and strengthening others. One of the midwives also expressed that the task of making others feel welcome was a privilege. The importance of the doulas was a topic that all the midwives talked about a lot. They highlighted their commitment several times and described how they contributed to creating an even more relaxed and supportive atmosphere by, among other things, praising the other women during the dance. One midwife claimed that it would not have been possible to carry out the project without the doulas, partly because of the linguistic matter, but also because of the many other positive factors they contributed to the project. The pregnant women expressed how the doulas made them feel more secure and how they valued their involvement. Even the doulas themselves experienced how the other women became calmer in their presence.

The midwives and doulas highlighted the importance of the project for the doulas, and how it might have had an important supportive impact on them privately. They had the perception that the project helped them develop not only in the role of doula but also on a personal level

I think that the doulas probably had been sitting at home for quite some time before they got this project. Not so many social occasions or the feeling of being a part of society. So, for them, this was probably amazing.

(Midwife)

#### Knowledge sharing

The pregnant women experienced the informative conversations as valuable and appreciated the comprehensive information that was given. They also described a great commitment to sharing knowledge from those who were responsible for the project. The content of the informal conversations was experienced as being quite fluid. One of the midwives explained that the exchanged information was quite spontaneous and adapted based on the pregnant women’s interests and the issue that came up at that specific time.

And I informed about what I had planned to inform about, but then it is not always—and I can experience that quite often—there may be cultural differences or so, that often you feel that this is what I want to inform about and this is what I think is important. And then they (the pregnant women) think of and ask about completely different things …

(Midwife)

The pregnant women expressed that they enjoyed discussing the different topics. One of them emphasized that she perceived those who were responsible as being very competent, with a lot of experience. The midwives also described how the pregnant women seemed to appreciate the informal part of the intervention. The doulas, in turn, emphasized that the informative conversations were one of the most important parts of the project. This was due to the fact that this group of women, who do not speak Swedish, are excluded from ordinary antenatal education groups. Knowledge sharing was a key to promoting equal health among women. Through participation in this targeting project, the pregnant women were able to take part in the parental education and childbirth preparation to which they were entitled and that they otherwise could have missed. This was confirmed by the pregnant women, who expressed that, as non-Swedish speakers, it was difficult to find information and that they appreciated the opportunity given.

And then it was good to get the information. […] I think it is important to have someone to talk to. To know where to turn for help, and so on. There is so much information, so it can be difficult, especially if you do not have Swedish as your first language.

(Pregnant woman)

#### Cultural exchange

Cultural exchange seemed to be an important part of the project for everyone involved, and it also seemed to arouse a lot of commitment. The group sessions were described as interesting and an opportunity to get greater insight into other people’s lives, as well as to broaden one’s horizons. Hence, such an opportunity to share different cultural experiences with each other was appreciated and approached with curiosity.

It was mixed. I am from a country in Africa and there were also Swedish women there. It was very, very, fun. For me, it was so much fun.

(Pregnant woman)

The importance of the cultural exchange was emphasized for both the foreign-born woman and the natives. The foreign-born women were given the opportunity to meet other people and to listen and talk in Swedish, which developed their language skills. At the same time, the meeting and cultural exchange were just as important for the natives. Getting outside of one’s comfort zone and experiencing another culture contributed to an increased understanding of other people.

We invite Swedish women as well, Swedish women also have the same interest in the project […] Welcome, welcome, welcome. That’s a great idea. When Swedish women also participate, we still talk with each other, we drink coffee together and sit together, and the Swedish women speak Swedish and the others try to listen and speak in English. We get closer to each other. That’s really good.

(Doula)

The doulas emphasized the importance for the community itself and described that taking part in this kind of intervention created a bridge for the midwives, allowing them to overcome social distance and giving them a chance to communicate without the barrier that often comes with the profession, thereby building trusting relationships, with pregnant women who did not speak any Swedish. This was confirmed by the participating midwives who expressed the benefit of the project, i.e., that it had given them greater insight and knowledge into other cultures and thereby developed their professional and cultural skills.

## Discussion

This study has presented the findings from a lifestyle intervention focusing on integration and physical activity, from the perspectives of all the participants: pregnant women, midwives, and cultural interpreter doulas. The main finding expressed by the participants was a strength, and a “woman” power, both individual and collective forms, which generated the experience of womanhood. The studied intervention formed a social space and gathered women from different groups of society with different backgrounds and ethnicities, with pregnancy being the common denominator. This social space created experiences of belonging, joy, and commitment. Our findings of the importance of getting together with other women during pregnancy are aligned with previous research (Barimani et al., 2018; Lupton, 2016; Modh et al., 2011). Connecting to and sharing experiences with other women during pregnancy is, according to Modh et al. (2011), part of the process of preparing for motherhood. Receiving social and professional support from others, such as the women in the present study, may facilitate the transition to motherhood (Barimani et al., 2018)

Additionally, the findings of this study indicate the positive experiences of both receiving and providing support to and from others. Being able to support others and share knowledge as well as experiences seemed to strengthen the participating women in the lifestyle intervention.

The importance of social support, a supportive environment, and a socially well-functioning network for the physical and mental health of pregnant women is also emphasized by Jalili Bahabadi et al. (2020). Moreover, in this study, the participants’ commitment seemed to grow when they realized that their contribution was valuable to others. Similar patterns have been found by Michalski et al. (2020), who described how belongingness and committedness can have positive effects on quality of life and, in turn, promote a sense of identity and confidence. Furthermore, the informative conversations in the lifestyle intervention seemed to resemble a participant-led female support group, which in a previous context, in combination with medical support, has been shown to have significant effects on both pregnant women’s and the unborn children’s health (Gabbe et al., 2017; Patel et al., 2018). A conclusion resulting from this can be that pregnancy groups that help build social support, like “Dancing for birth,” may result in positive health outcomes. The pregnant women in the intervention also described the value of the commitment and the support from the doulas as well as the midwives, and how it made them feel safe. Previous research highlights how female leaders from other ethnicities can function as important role models for foreign-born women, which could benefit their own integration process (Msengi et al., 2015). Similar findings have been found in another study, with the aim of investigating doula-based interventions (Akhavan et al., 2015).

Being part of a group and sharing experiences with others in similar situations was significant. It seemed to increase feelings of inclusion and create new opportunities for social interaction. Moreover, the experience of being included in a female social network was described as valued by the pregnant women. Connections and discussions with peers during pregnancy are valued (Entsieh & Hallstrom, 2016) and Lupton (2016) emphasized that the main reason why many pregnant women turn to different social media platforms and discussion forums online is to connect with other women.

The findings of this study indicate that the participants, pregnant or not, and regardless of origin, indicated that the intervention had a positive effect on both their mental and physical health. The participants appreciated the opportunity to get their bodies moving and emphasized the joy of dancing. Similarly, previous research describes dance as a joyful, relaxing, and strengthening activity, which has also been shown to benefit the connection between the expectant mother and her unborn child as well as mental well-being during pregnancy (Jackson, 2015). A connection between social support and regular physical activity during pregnancy has also been found (Morris et al., 2020; O’Brien et al., 2017; Warren et al., 2017). However, barriers to physical activities during pregnancy are described, for example, a lack of information about safe activities and not having the physical opportunity to carry out physical activity (Flannery et al., 2018; Warren et al., 2017). Hultstrand et al. (2020) described foreign-born women as particularly vulnerable when it comes to barriers to participation in physical activity. Hence, it is of utmost importance to focus on health-promoting lifestyle interventions targeting physical activity in pregnancy, such as “Dancing for birth,” which includes and encourages physical activity among foreign-born women.

Important findings in this study were that the intervention “Dancing for birth” contributed to integration as well as increased the women’s feeling of belonging. Previous research has established belongingness contributes to the promotion of health, and to feel belonging to people from both the host country and the country of origin has been shown to benefit the health of the foreign-born individual (Seppänen et al., 2020. However, the given opportunity to integrate into the lifestyle intervention was found to benefit not only those with a foreign background but also everyone involved. This created a unique opportunity for cross-cultural learning experiences, which led to a greater understanding for everyone involved. Previous research confirms that multicultural women support groups can both strengthen women and increase their sense of well-being, but also serve as a foundation for integration into the new society (Msengi et al., 2015). The Public Health Agency of Sweden (2020) emphasized the importance of creating opportunities for different groups in society to meet and interact with each other, to increase equality in health. Accordingly, and in view of the results of this study, lifestyle interventions like “Dancing for birth” should be given more priority to promote health among women.

### Study limitations and strengths

This study has both limitations and strengths. Although qualitative research has a more forgiving view of sample size, the small number of participants is a limitation (Nowell et al., 2017). This also includes a limitation in the intervention with the low number of new contacts for the participants in the lifestyle intervention, which could have been much larger. However, it has been found that the foreign-born as a group, and especially women, are more difficult to recruit for research (Cudjoe et al., 2019). For that reason, this study is particularly important, as it contains experiences from a part of the population that otherwise are not usually heard to the same extent. The fact that all participants could express themselves, regardless of language difficulties, can be a strength, as many studies choose to exclude participants who do not speak Swedish. By describing different perspectives on the intervention, including the experiences of pregnant women as well as of the doulas and the midwives, foreign-born as well as natives, a variation has been produced. According to Nowell et al. (2017), this can also strengthen the credibility and transferability of the study.

Additionally, the protracted data collection can be considered another limitation of the study. Nowell et al. (2017) emphasized the risk of memory bias, which was something that some of the participants themselves expressed. The fact that the interviews were conducted and transcribed by three different authors can, incidentally, also be considered a limitation, as there is a risk that it negatively affects the credibility (Nowell et al., 2017). However, on the other hand, involving several authors in the analysis process can strengthen the result, as more perspectives are generated, and investigator triangulation is achieved.

## Conclusions and implications for practice

The findings of this study indicate that the lifestyle intervention “Dancing for birth” provided positive shared health-promoting experiences among the participants with social inclusiveness and a commitment to supporting each other. This seemed to encourage the sense of strength as a woman—a strength both for the individual woman and as a and a kind of collective women’s power with social inclusiveness. Given the World Health Organization’s (2016) implications of antenatal care, it is necessary to prioritize and promote positive pregnancy experiences and identify and implement group-based programmes with health-promoting interventions to improve the quality of antenatal care. With that in mind, the findings in this study suggest that antenatal care may be improved by health innovative tailored interventions such as “Dancing for birth,” to foster equal and safe healthcare during pregnancy.
